# The role of inhibition of osteocyte apoptosis in mediating orthodontic tooth movement and periodontal remodeling: a pilot study

**DOI:** 10.1186/s40510-021-00366-4

**Published:** 2021-07-26

**Authors:** Michele Kaplan, Zana Kalajzic, Thomas Choi, Imad Maleeh, Christopher L. Ricupero, Michelle N. Skelton, Madeleine L. Daily, Jing Chen, Sunil Wadhwa

**Affiliations:** 1 Division of Orthodontics, College of Dental Medicine, Columbia University, New York, NY USA; 2 Department of Oral Health and Diagnostic Sciences, Division of Oral Medicine, UConn Health, Farmington, CT USA

**Keywords:** Orthodontic tooth movement, Osteocyte apoptosis, Bisphosphonate

## Abstract

**Background:**

Orthodontic tooth movement (OTM) has been shown to induce osteocyte apoptosis in alveolar bone shortly after force application. However, how osteocyte apoptosis affects orthodontic tooth movement is unknown. The goal of this study was to assess the effect of inhibition of osteocyte apoptosis on osteoclastogenesis, changes in the alveolar bone density, and the magnitude of OTM using a bisphosphonate analog (IG9402), a drug that affects osteocyte and osteoblast apoptosis but does not affect osteoclasts.

**Material and methods:**

Two sets of experiments were performed. Experiment 1 was used to specifically evaluate the effect of IG9402 on osteocyte apoptosis in the alveolar bone during 24 h of OTM. For this experiment, twelve mice were divided into two groups: group 1, saline administration + OTM_24-h_ (n=6), and group 2, IG9402 administration + OTM_24-h_ (n=6). The contralateral unloaded sides served as the control. The goal of experiment 2 was to evaluate the role of osteocyte apoptosis on OTM magnitude and osteoclastogenesis 10 days after OTM. Twenty mice were divided into 4 groups: group 1, saline administration without OTM (n=5); group 2, IG9402 administration without OTM (n=5); group 3, saline + OTM_10-day_ (n=6); and group 4, IG9402 + OTM_10-day_ (n=4). For both experiments, tooth movement was achieved using Ultra Light (25g) Sentalloy Closed Coil Springs attached between the first maxillary molar and the central incisor. Linear measurements of tooth movement and alveolar bone density (BVF) were assessed by MicroCT analysis. Cell death (or apoptosis) was assessed by terminal dUTP nick-end labeling (TUNEL) assay, while osteoclast and macrophage formation were assessed by tartrate-resistant acid phosphatase (TRAP) staining and F4/80+ immunostaining.

**Results:**

We found that IG9402 significantly blocked osteocyte apoptosis in alveolar bone (AB) at 24 h of OTM. At 10 days, IG9402 prevented OTM-induced loss of alveolar bone density and changed the morphology and quality of osteoclasts and macrophages, but did not significantly affect the amount of tooth movement.

**Conclusion:**

Our study demonstrates that osteocyte apoptosis may play a significant role in osteoclast and macrophage formation during OTM, but does not seem to play a role in the magnitude of orthodontic tooth movement.

## Introduction

In long bones, bone mass and architecture are determined primarily by the mechanical loading environment, whereby changes to this environment cause the bone trabeculae and cortex to remodel, accordingly [1, 2]. Increased mechanical strains often induced by physical activity results in increased bone mass [3]. On the other hand, decreased strain magnitude from activities such as prolonged bed rest leads to bone loss [4]. Mechanical loading influences the osteocytes, the mechanosensing cells within the bone [5]. Osteocytes form a canalicular-lacunar network that allows their communication with other osteocytes, osteoblasts, and osteoclast progenitor cells [6]. Mechanical loading-induced fluid flow through the canalicular-lacunar network provides nutrients to osteocytes and results in an upregulation of anabolic factors [7]. In contrast, loss of loading causes a decrease in fluid flow and an increase in osteocyte apoptosis. Apoptotic osteocytic bodies have been shown to release potent factors that cause an increase in osteoclastogenesis [8–10].

Similar to long bones, the alveolar bone (AB) that houses the dentition acclimates to changes in mechanical loading. External orthodontic forces applied to teeth change the mechanical loading environment of the alveolar bone and subsequently elicit a cellular response that leads to bone adaptation into a new functional environment. Recently, it has been shown that osteocytes play a crucial role in orthodontic tooth movement (OTM) [11–13] and that orthodontic forces cause an increase in osteocyte apoptosis that peaks before the appearance of osteoclasts [14]. Therefore, studies focused on inhibiting osteocyte apoptosis may provide insight on the biological mechanism of osteoclastogenesis during orthodontic tooth movement.

Bisphosphonates have been shown to induce osteoclast cell death and to inhibit osteoblast and osteocyte apoptosis [15]. Recently, a bisphosphonate analog (IG9402) has been developed that effectively blocks osteocyte and osteoblast apoptosis both in vitro and in vivo, but has no effect on osteoclasts [16]. The use of IG9402 has been shown to prevent mechanical unloading induced osteocyte apoptosis in long bones [17]. Therefore, the goal of this study is to analyze the effect of IG9402 (osteocyte apoptosis inhibitor) on orthodontic tooth movement. We hypothesize that inhibition of osteocyte apoptosis will affect osteoclastogenesis and cause a subsequent decrease in the amount of orthodontic tooth movement.

## Materials and methods

### Study design and application of orthodontic appliance

All experiments were performed under an institutionally IRB-approved IACUC protocol for the use of animals in research (Columbia University AC-AAAY3451). In total, thirty-two CD-1 male mice (12–14 weeks old) were used in this study. Mice were divided into either 24-h OTM groups (n=12) or 10-day OTM groups (n=20). IG9402 was a kind gift from Gador (Buenos Aires, Argentina). It has been previously reported that 0.60 mg/kg/day of IG9402 injected subcutaneously daily causes inhibition of osteocyte apoptosis [16]. Therefore, a stock solution of IG9402 of 0.2 mg/ml was made. The volume of the stock solution injected subcutaneously in the mice was determined by body weight (weight of mice (g) × 3 μl of stock solution). Mice were administered daily subcutaneous injections of IG9402 or the equivalent volume of saline for 24 h or 10 days.

#### To assess whether OTM-induced osteocyte apoptosis can be blocked by IG9402

**Fig. 1:**
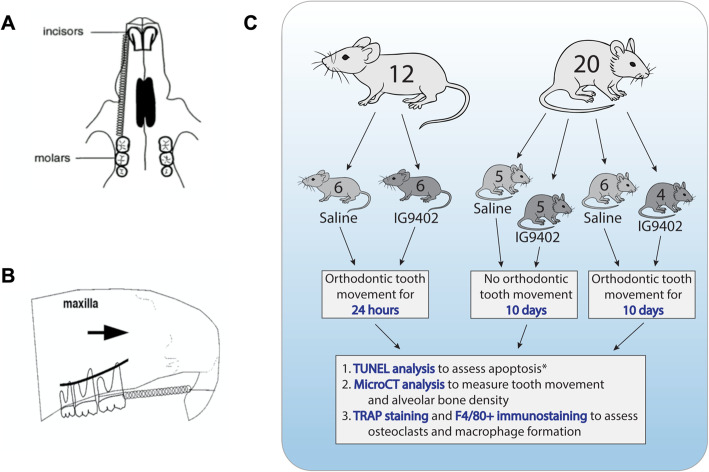
Illustration of the tooth movement model used in this study. **A** The placement of NiTi coil spring in the orthodontic tooth movement (OTM) groups. **B** The spring was activated from the maxillary first molar to the central incisor for 24 h (left side) or 10 days (right side). Arrow shows the direction of the orthodontic force. **C** Photo summarizing study design. Twenty-four-hour OTM samples were only stained with TUNEL for apoptosis analysis

Twelve male CD-1 mice (12–14 weeks old) were divided into two groups. All mice were treated for tooth movement for 24 h with Ultra Light (25 g) Sentalloy Closed Coil Springs (Densply GAC, Cat # 10-000-26) according to the tooth movement protocol previously developed [14, 18] (Fig. 1A, B). The eyelets of the springs were removed from both ends of the springs, and springs were attached and activated between the left first maxillary molar and the central incisor with 0.004″ ligature tie. The ligature ties were secured with flowable composite. At the time of loading, mice in group 1 (n=6) were subcutaneously injected with saline (saline + OTM_24-h_). Mice in group 2 (n=6) were subcutaneously injected with IG9402 (0.60 mg/kg/day) (IG9402 + OTM_24-h_). The contralateral unloaded sides served as the control. Mice were sacrificed 24 h after initiation of OTM.

#### To assess the role of osteocyte apoptosis in mediating the magnitude of orthodontic tooth movement

Twenty male CD-1 mice (12–14 weeks old) were divided into 4 groups. OTM protocol was the same as the 24-h experiment, but the spring was placed on the right side for 10 days (Fig. 1A, B). Group 1 (n=5), saline administration without OTM; group 2 (n=5), IG9402 administration without OTM; group 3 (n=6), saline + OTM_10-day_; and group 4 (n=4), IG9402 + OTM_10-day_. We had initially planned for n=6 for IG9402 + OTM_10-day_ group; however, two mice had a slight distortion with the orthodontic springs and therefore were not included in the study. Mice were administered daily subcutaneous injections of IG9402 (0.60 mg/kg/day) or the equivalent volume of saline for 10 days. The spring attached to the incisor was adjusted when necessary to accommodate the continuous incisor growth. Mice were sacrificed 10 days after initiation of OTM.

All experimental mice were fed a soft dough diet (Transgenic Dough Diet; Bioserv, Frenchtown, NJ) during the experimental period. After completion of the experiment, the mice were euthanized with CO2 followed by cervical dislocation. The maxillae were harvested and fixed in 10% formalin for 7 days.

### MicroCT analysis

**Fig. 2:**
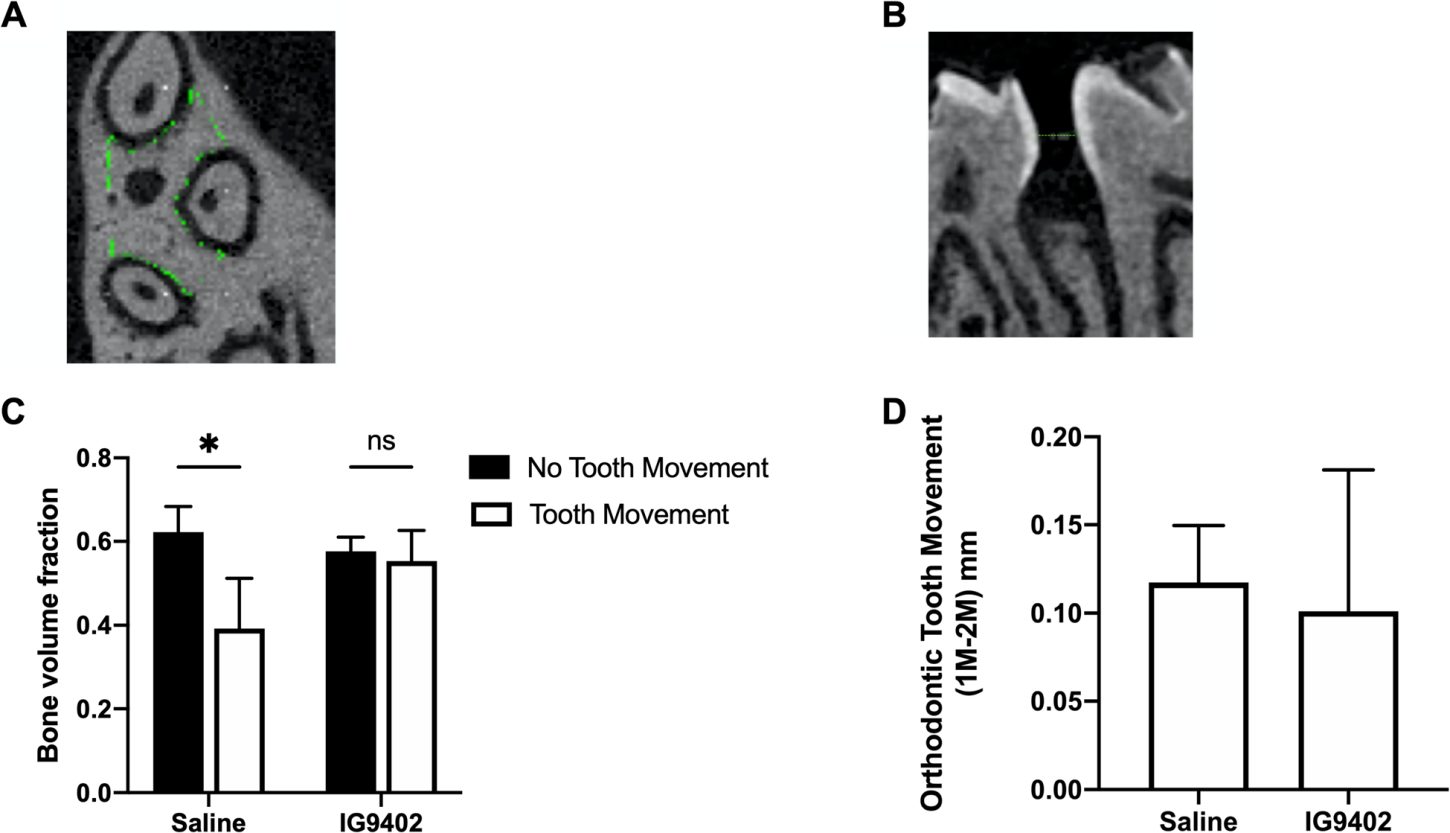
Micro-CT analysis. **A** Region of interest (ROI) for alveolar bone analysis (BVF) extended from the mesial side of the distobuccal root to the distal side of the mesiobuccal root of the right maxillary molars (traced area). **B** Linear measurements of tooth movement were obtained from the intermolar distances (HOC-HOC) on the MicroCT 2D image slice of hemisected maxilla. **C** Graph showing the comparison of alveolar bone densities (BVF) between experimental groups. BVF bone volume fraction (bone volume divided by total volume). Note the significant decrease in BVF by saline + OTM_10-day_-treated groups (unpaired t-test, p<0.02). OTM caused no significant difference in BVF in the IG9402 + OTM_10-day_-treated group (unpaired t-test, p=0.25). Each value represents the mean ±SD (n=4-6). **D** Graph showing the intermolar distances (HOC-HOC) measured for IG9402 + OTM_10-day_- and saline + OTM_10-day_-treated groups. Each value represents the mean± SD (n=4-6). No significant difference was detected in OTM between IG9402-treated and saline-treated groups (unpaired t-test, p=0.6650)

After 10 days of orthodontic tooth movement the maxillae including maxillary molars were scanned in a VivaCT 80 system using energy settings of 55 kV, 109 μA. 3D images were reconstructed to yield a 10.4-μm isotropic voxel size. The region of interest chosen for the bone parameter analysis was the furcation area of the maxillary first molars extending from the distal side of mesial root to the mesial side of distobuccal root (Fig. 2A) at the first slice that showed three distinct root structures until the last slice that showed three distinct root structures. Bone volume fraction (BVF) was calculated as the percentage of bone volume (BV) to total volume (TV) [19–21].

For each mouse, linear measurements of tooth movement were assessed by the intermolar distances from the distal height of contour (HOC) of the first molar crown to the mesial HOC of the second molar crown from three adjacent 2D sagittal MicroCT images on the plane that revealed the most root structure (Fig. 2B) and their mean value was used for orthodontic tooth movement distance. All measurements were made by one observer.

### Tissue preparation for histologic analysis and TRAP staining

Maxillae were demineralized in 14% EDTA, PH 7.1 for 4 weeks at 4 °C and then processed for paraffin embedding (Paraplast PLUS, Fisher Scientific). Five-micrometer serial sagittal sections were cut using the Leica microtome RM 2125R7 (Leica Microsystems, Nussloch GmbH). Ten to 20 mid-sagittal root sections of first maxillary molars were examined using the Zeiss Axiovert 200 Microscope and digital camera. Histological analysis was performed on the compression side (mesial side of the distobuccal root of first maxillary molars). Three sections that revealed the most root and pulp structure and the 5–10 images adjacent on either side were chosen for analysis. For semi-quantitative analysis (apoptosis), the value from 3 sections was averaged for each mouse and for non-quantitative analysis (H&E, F4/F80+, and TRAP), the image that was most representative was chosen for the presentation.

For histological analysis, sections were stained with routine hematoxylin and eosin (H&E) stain. To visualize osteoclasts, sections were stained for tartrate-resistant acid phosphatase (TRAP) activity using the Leukocyte Acid Phosphatase Kit (Sigma-Aldrich, St Louis, MO) according to the manufacturer’s instructions [18] and visualized similarly to the protocol previously established [19, 21].

### TUNEL assay

To visualize apoptosis, TUNEL staining was performed according to the manufacturer’s instructions using the Promega DeadEnd™ Fluorometric TUNEL System # G3250 [14, 22]. The fluorescein-12-dUTP-labeled DNA was visualized by Leica DM500 B Microscope and digital camera. DAPI nuclear counterstaining was performed to visualize total cells. Osteocyte and PDL apoptotic fluorescent cells were analyzed using a custom ImageJ macro. Raw images were converted to binary images using the Intermodes threshold, and individual nuclei were counted using the built in Analyze Particles function. TUNEL-positive cells were counted on the compression side of alveolar bone and PDL (mesial surface of the distobuccal root) and normalized per number of DAPI-positive cells in the same tissue section. The ratio of cells was calculated by one observer for each section as TUNEL-positive cells/total cells in both the AB and PDL. The ratio from three sections was then averaged for each mouse. Sixteen randomly selected measurements were repeated with an intra-class coefficient of r = 0.9998. The averaged values were used to conduct statistical analysis.

### Immunohistochemistry

Immunohistological analyses for the macrophage marker F4/80^+^ were performed. Deparaffinized sections were incubated with 0.3% hydrogen peroxide (H_2_0_2_) for 25 min. Heat-mediated antigen retrieval was done by immersing the slides in 1x Citrate Buffer pH 6.0 at 60 °C overnight. The following day, sections were blocked with 10% normal goat serum for 2 h and incubated with rabbit anti F4/80^+^ polyclonal antibody (Thermo Fisher Scientific, Rockford, IL, PA5-32399) at a concentration of 1:250 overnight at +4 °C. The next day, tissues were incubated with a goat biotinylated secondary antibody and the signal was developed with a biotin/avidin system. The secondary antibody and developing reagents (DAB) were obtained from Vector Laboratories (Burlington, CA) and included in the Vector Elite ABC kit (PK6101). Negative controls were performed by omitting the primary antibody.

### Statistical analysis

Statistical analyses were carried out using GraphPad Prism (GraphPad Software, INC, version 8.4.3 for Mac OS X, San Diego, CA, USA). Normality of distribution was confirmed prior to analysis. Osteocyte and PDL apoptosis in IG9402 + OTM_24-h_ was compared to saline + OTM_24-h_. Tooth movement measurements from IG9402 + OTM_10-day_ were compared to the measurements from saline + OTM_10-day_. BVF measurements from saline + OTM_10-day_ and IG9402 + OTM_10-day_ were compared to their respective unloaded control samples. All normally distributed data was analyzed with unpaired t-test, and data without normal distribution was analyzed with Mann-Whitney test. Significance was accorded P <0.05.

## Results

### Orthodontic induced osteocyte apoptosis is blocked by IG9402

**Fig. 3:**
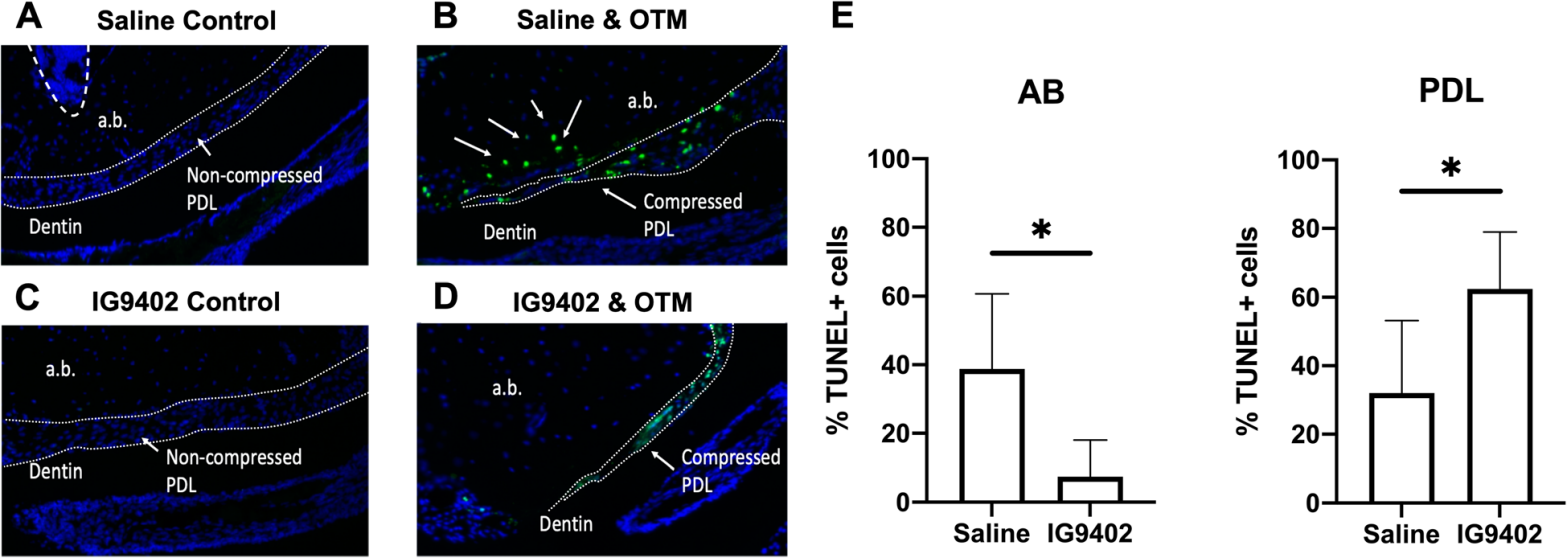
Evaluation of apoptosis on the compression area in experimental groups (**B**, **D**) with TUNEL labeling. Apoptotic cells are labeled green (arrows) and total cells (nuclei) are labeled blue. PDL and hematopoietic marrow are outlined with a white dashed line on images **A**–**D**. Note decreased number of alveolar apoptotic cells in orthodontically loaded maxillae treated with IG9402 + OTM_24-h_ (**D**) compared to the saline + OTM_24-h_-treated group (**B**). However, IG9402 + OTM_24-h_ has increased apoptosis in the compressed PDL (**D**). **E** Graph showing the comparison of apoptotic cells (TUNEL +) in the AB and PDL following OTM. Saline + OTM_24-h_ has a statistically significant increase in apoptosis in the alveolar bone compared to the IG9402 group (Mann-Whitney p<0.0001). However, IG9402 + OTM_24-h_ has a statistically significant increase in apoptotic cells in the PDL (unpaired t-test, p=0.0196). Each value represents the mean ±SD (n=6)

We found a significant decrease in apoptosis of osteocytes on the compression side of the alveolar bone (Mann-Whitney p<0.0001) in IG9402 + OTM_24-h_ group (mean 7.50 ± 10.56), compared to saline + OTM_24-h_ (mean 38.77 ± 21.91), suggesting that IG9402 is successful in inhibiting OTM-induced osteocyte apoptosis in the alveolar bone 24 h after force application (Fig. 3).

Interestingly, there was a significant increase in apoptotic cells within the PDL (unpaired t-test, p=0.0196) when IG9402 + OTM_24-h_ group (mean 62.49 ± 16.50) was compared to saline + OTM_24-h_ (mean 32.08 ± 21.16).

Unloaded contralateral controls had no osteocyte or PDL apoptosis and were not included in statistical analysis.

### Inhibition of osteocyte apoptosis does not affect 10-day magnitude of tooth movement but prevents orthodontic induced decrease in alveolar bone density

**Fig. 4:**
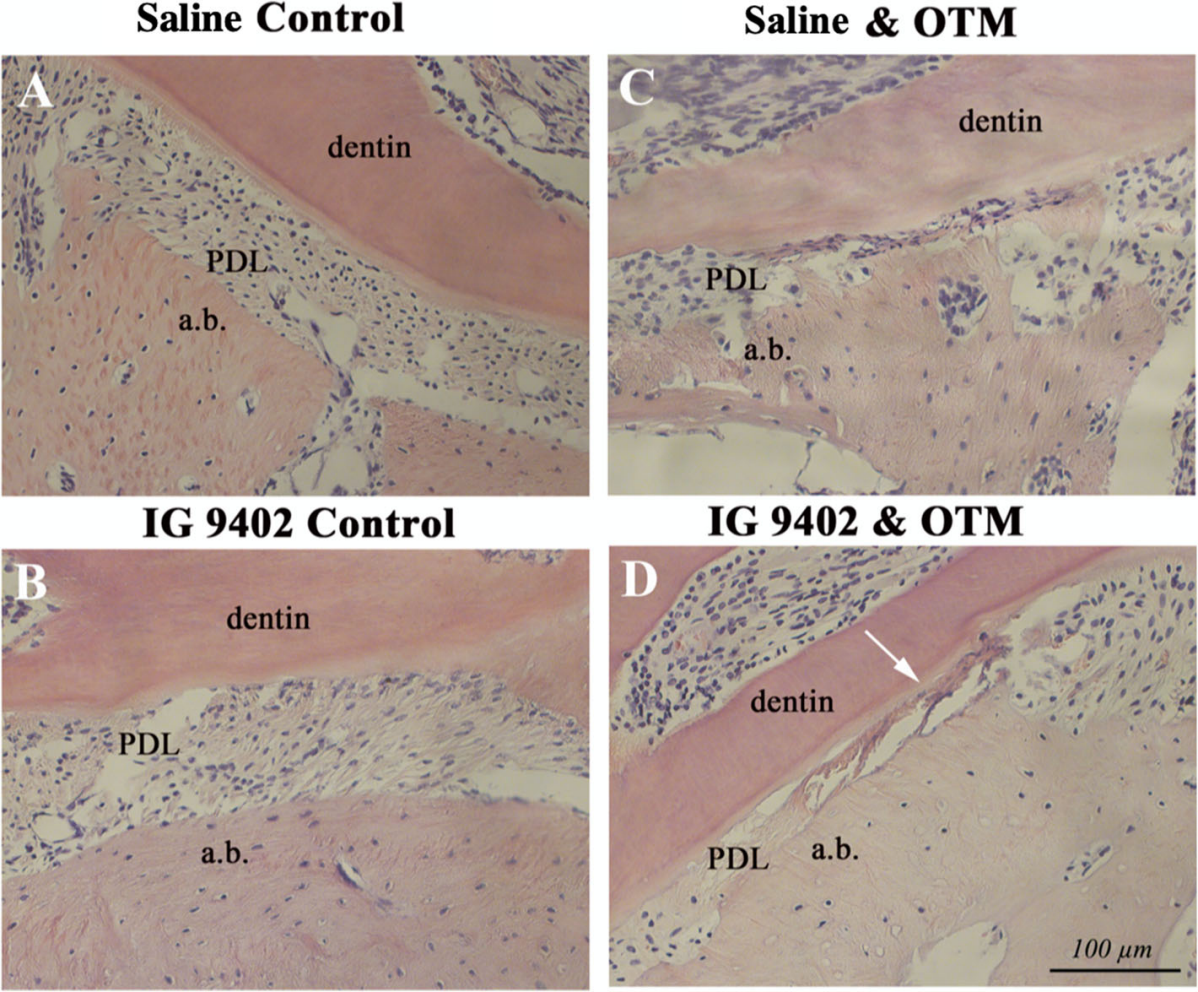
Histological analysis of the compression area in experimental groups (hematoxylin/eosin staining). Non-compressed PDL was observed in the control groups (**A**, **B**). Significant PDL compression was observed in orthodontically loaded groups (**C**, **D**). Note extensive PDL acellular areas in orthodontically loaded maxillae treated with IG9402 + OTM_10-day_ (arrow) (**D**)

In both experimental groups, IG9402 + OTM_10-day_ and saline + OTM_10-day_, PDL space on the mesial side of the distobuccal root in loaded molars was significantly compressed. However, in the IG9402 + OTM_10-day_ group there, was a noticeably larger eosinophilic area within the compressed ligament accompanied by accumulation of extensive, still acellular, amorphous material. This suggests that the area of hyalinization, which normally occurs on the compression side of PDL in very early phases of OTM and later is resorbed by macrophages, is still present and not sufficiently removed with IG9402 treatment if compared to saline treatment (Fig. 4).

**Fig. 5:**
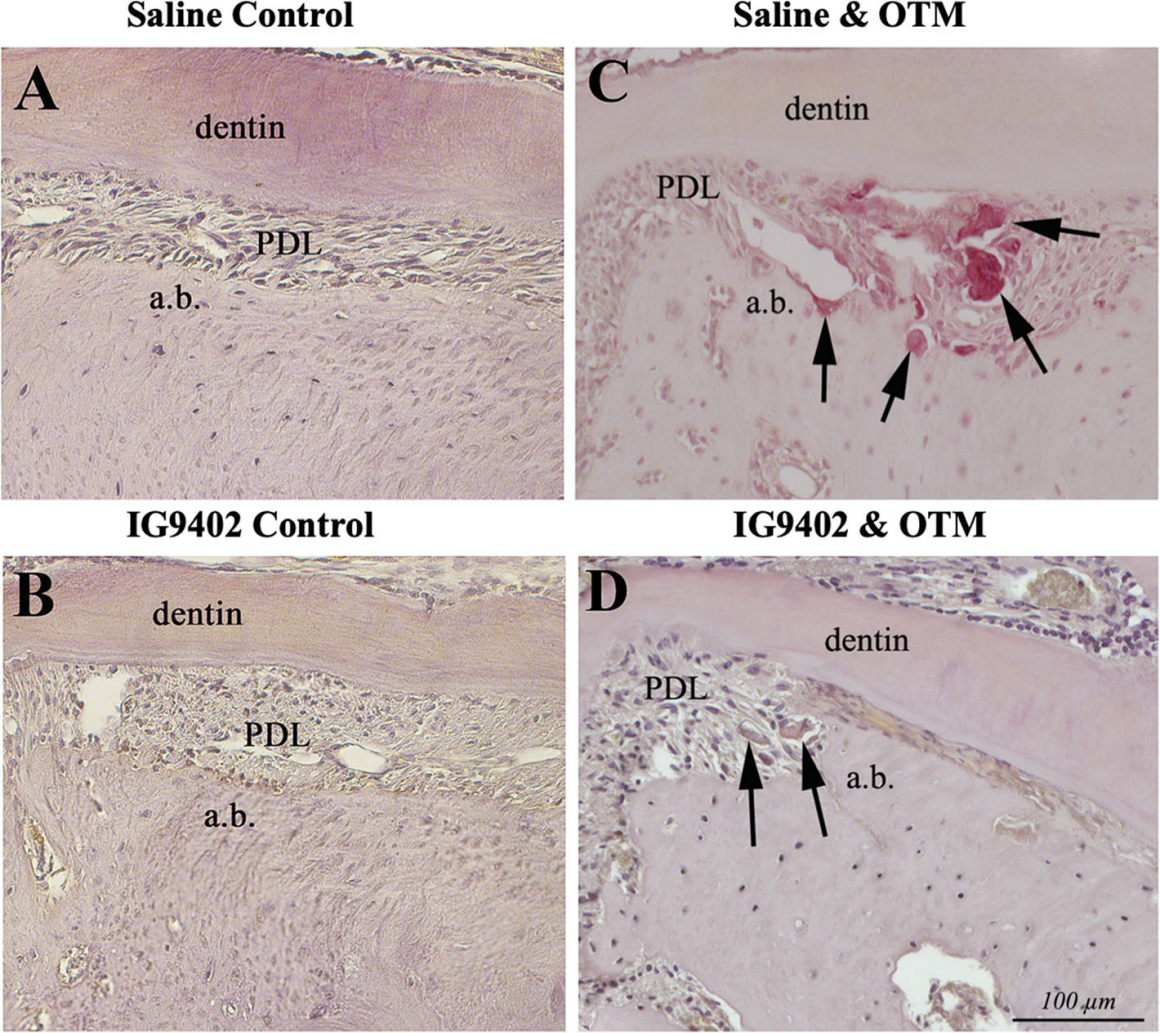
Histological examination of osteoclasts on the compression area in experimental groups. No osteoclasts were seen in orthodontically unloaded groups (**A**, **B**). Many multinucleated cells were detected within the PDL, on the alveolar bone and dentin surface in both loaded groups (**C**, **D**), but with IG9402 treatment, multinucleated cells were not stained intensively red; they were TRAP negative

Osteoclasts were monitored on the compression side on the alveolar bone and dentin [19–21]. They were barely detected in unloaded controls. But, sections from orthodontically loaded groups revealed numerous multinucleated, osteoclast looking cells (Fig. 5C, D). In saline + OTM_10-day_ group, these cells were stained intensively red (TRAP positive) and often exhibited multiple nuclei in their cytoplasm (>3) confirming their activity. In the IG9402 + OTM_10-day_ group, osteoclasts had atypical morphology, seemed to be smaller in size, stained brownish-yellow (instead of intensively red), and rarely exhibited more than 2 nuclei in the cytoplasm (Fig. 5D). This observation suggests that terminal differentiation of osteoclasts (that is confirmed with the strong red staining using this TRAP method) might be severely affected by the application of IG9402.

**Fig. 6:**
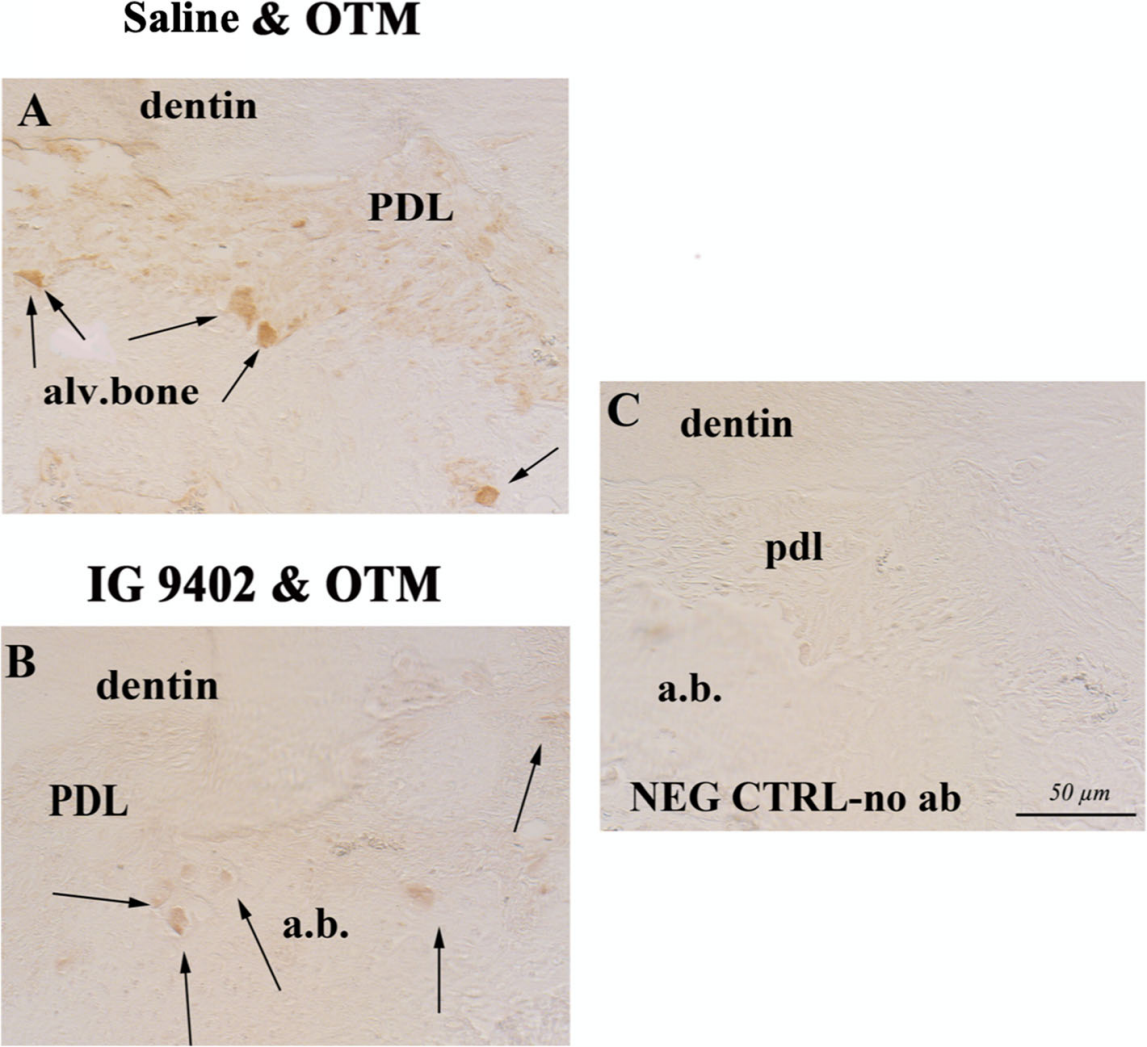
Immunostaining on macrophage marker F4/80^+^ on the compression side. There appeared to be an increased number of macrophages (stained brown) in orthodontically loaded group treated with saline (**A**) compared to orthodontically loaded group treated with IG9402 (stained less intensively) (**B**) (arrows). **C** Negative control, primary antibody omitted

Similar to osteoclasts, macrophages were not detected in the control samples not undergoing OTM. However, there appeared to be a weaker staining on F4/80^+^ macrophage marker on the compressed side in the IG9402 + OTM_10-day_ group (Fig. 6) compared to the saline + OTM_10-day_ group.

MicroCT analysis showed a significant decrease in the alveolar bone density (BVF) of loaded first maxillary molars in the saline + OTM_10-day_ group compared to the respective contralateral unloaded control (unpaired t-test, p<0.02). However, no significant decrease was seen between loaded maxillary molars of the IG9402 + OTM_10-day_ group and their respective unloaded control (unpaired t-test, p=0.25), suggesting that osteopenia that normally occurs during the tooth movement does not occur with the application of IG9402 (Fig. 2C).

The intermolar distances in unloaded controls were measured 0 mm and were not included in statistical tests. In the IG9402 + OTM_10-day_ group, the distance was slightly lower (mean=0.101mm ± 0.080) than in the saline + OTM_10-day_ group (mean= 0.117mm ± 0.032). However, it is not statistically significant (unpaired t-test, p= 0.6650) (Fig. 2D).

## Discussion

We and others have shown that orthodontic forces cause a significant increase in osteocyte apoptosis and that its peak (24–48 h of OTM) precedes the appearance of osteoclasts [14, 23]. Osteocytes are deeply embedded in mineralized matrix and are not accessible by scavengers that phagocytose cells undergoing apoptosis and prevent inflammation. We posit that orthodontic forces cause a decrease in canalicular fluid flow [12, 24] and an increase in alveolar bone microcracks [25]. The lack of fluid flow leads to decreased nutrient supply, while the microcracks damage the osteocytic network, which both influence osteocytes to undergo apoptosis. Since not accessible by scavengers, dying osteocytes release factors through the canalicular-lacunar network that cause other viable osteocytes to increase their expression of mediators important for macrophage and osteoclast formation and the removal of hyalinized PDL. Therefore, when they undergo apoptosis, osteocytic cytoplasmic membrane ruptures causing a response known as secondary necrosis [26] that releases factors that travel through the canalicular network and causes the initiation of macrophage and osteoclast formation [27].

Recent research implied the alveolar bone is involved in the biology of OTM. A study showed that osteocyte specific deletion of RANKL inhibited OTM by 50% [28]. However, traditionally, it was believed that the PDL is the principle mediator in the biology of OTM [29, 30]. The PDL contains many cells predominantly fibroblasts, osteoblasts, macrophages, and immune and mesenchymal cells [31]. When excessive compressional or stretching forces have been applied to the PDL during OTM, it results in either cell death within the PDL, increased RANKL expression, and/or a local inflammatory response, which in turn causes the recruitment of phagocytic cells. It has been shown that PDL cells adjacent to hyalinized PDL and alveolar bone on the compression side during OTM in mice presented substantial expression of vascular endothelial growth factor (VEGF), an important factor for angiogenesis [32]. The renewal of the vascular supply in compressed PDL contributes to osteoclasts recruitment and differentiation and has been an important process for healing and remodeling of the periodontium [32]. Interestingly, we found that IG9402 inhibited osteocyte apoptosis in the alveolar bone; however, at the same time cell apoptosis within the PDL was statistically increased. We also found that after 10 days of OTM there was a nonsignificant decrease in the amount of tooth movement in the IG9402-treated group compared to the saline-treated group. This may suggest that PDL and osteocyte apoptosis are equally important, not mutually exclusive, and have the ability to compensate for each other in the biology of OTM. However, in order to confirm whether there is a true difference in OTM between the two groups, more animals are needed in future studies.

Classically, orthodontic tooth movement curve has three phases [33]. The first is initial displacement due to PDL deformation. The second is the lag phase in which there is no tooth movement but hyalinization. There is recruitment of scavenger cells allowing the teeth to move only after the removal of necrotic PDL. The third is the tissue turnover phase in which there are numerous resorptive cells and a relative continuous tooth movement [33]. Therefore, another possible explanation for nonsignificant change in the magnitude of OTM could be that the IG9402-treated group is still in the lag phase (confirmed by the presence of hyalinized areas) whereas the saline-treated group is just beginning the phase of continuous OTM. Therefore, during a longer OTM duration, there would be a significant difference in the OTM distances between the IG9402 + OTM_10-day_ group compared to the saline + OTM_10-day_ group.

Here, we found that OTM induces formation of mature osteoclasts and macrophages and increased bone resorption. OTM in mice treated with IG9402 caused no change in BVF, impaired macrophage formation, and formed immature osteoclasts. Our study shows that inhibition of osteocyte apoptosis affects OTM-induced osteoclastogenesis and macrophage formation and that it might lengthen the lag phase of OTM. This confirms that OTM-induced osteocyte apoptosis may be responsible for recruitment and proper differentiation of osteoclasts and macrophages. However, it leads to the question that without proper macrophages and osteoclasts, how is tooth movement occurring in the IG9402 group? We do not know the exact answer, but one possible explanation is that the hyalinized PDL can also be removed by secreted proteases from viable periodontal ligament cells [34].

It may be possible that the bisphosphonate analog IG9402 directly promoted osteoclast apoptosis, thus inhibiting osteopenia during tooth movement. In a number of studies, it was shown that IG9402 had no direct effect on osteoclasts [16]. In addition, if the drug had a pro-apoptotic effect on osteoclasts, we would still probably be able to find some maturing osteoclasts capable to reach terminal differentiation (characterized by intensively red-stained cytoplasm using commercial TRAP stains, and accompanied by multiple nuclei). Osteoclasts in this case would probably be presented with a lower number rather than the lower intensity of stain and marker used. In our experiments, we observed “osteoclast like” brown, immature looking, “TRAP very weak,” or even “TRAP negative cells.” None of the osteoclasts in our IG9402 samples showed normal morphology or intensively red-stained cytoplasm in histological sections.

Knowing the fact that the presence of TRAP enzyme in the cytoplasm of osteoclast is used as a marker for their full differentiation, polarity, and full level of activity, here we would assume that developing osteoclasts lack critical growth factors or nutrients needed for their terminal differentiation and these normally arrive from osteocytic network during the process of osteocyte apoptosis.

According to data from this study, the use of IG9402 may be beneficial for patients undergoing orthodontic treatment who have osteopenia. It has been shown that orthodontic tooth movement causes a decrease in alveolar bone density that is further exacerbated by increased age [35] and estrogen deficiency [36]. Since there has been a dramatic increase in the number of older patients seeking orthodontic treatment, the use of IG9402 in conjunction with OTM may be beneficial in preventing excessive bone loss particularly in post-menopausal women seeking comprehensive orthodontic treatment.

The limitations of this study include a small sample size. Further studies with a larger sample size are necessary to evaluate the effect osteocyte apoptosis inhibition has on the magnitude of OTM. Future experiments should be done with pan caspase inhibitors that will block apoptosis in the PDL and osteocytes in the AB to help delineate the specific role PDL apoptosis has on tooth movement compared to osteocytes. Due to the high prevalence of apoptosis found in the PDL in the IG9402 group, perhaps other compensatory mechanisms are occurring such as autophagy, paraptosis, mitotic catastrophe, or necrosis. In the PDL, it is unclear whether the cells are undergoing necrosis or apoptosis because TUNEL stains both processes. More studies are needed in order to specify the type of compensatory mechanism that occurs in the PDL when osteocyte apoptosis is inhibited. Another limitation is that in our study we used a Ni-Ti closed spring delivering 25 g of force between the first molar and incisor, which is high and may have caused extrusion of the molar. Future studies with Ni-Ti coil springs that deliver <10 g of force are planned.

## Conclusion

- IG9402 is an inhibitor of osteocyte apoptosis in the alveolar bone.
- PDL apoptosis may be just as important for OTM as osteocyte apoptosis in the alveolar bone.
- The inhibitor of osteocyte apoptosis (IG9402) severely affected osteoclast morphology and differentiation, formation of macrophages, and alveolar bone resorption during OTM.
- Future studies with multiple time points and other osteocyte apoptosis inhibitors, as well as a detailed analysis of mediators involved in this process, are needed to further clarify a role of osteocyte apoptosis in mediating OTM.

## Data Availability

All data generated or analyzed during the current study are included in this published article.

## Abbreviations

OTM: Orthodontic tooth movement
AB: Alveolar bone
PDL: Periodontal ligament
BVF: Bone volume fraction
BV: Bone volume
TV: Total volume of the region of interest
TRAP staining: Tartrate-resistant acid phosphatase staining
TUNEL: Terminal dUTP nick-end labeling
H&E stain: Hematoxylin and eosin
HOC: Height of contour
